# Surface stresses in complex viral capsids and non-quasi-equivalent viral architectures

**DOI:** 10.1098/rsif.2020.0455

**Published:** 2020-08-05

**Authors:** Giuliana Indelicato, Paolo Cermelli, Reidun Twarock

**Affiliations:** 1 Department of Mathematics, University of York, York, UK; 2 Department of Biology, University of York, York, UK; 3 Dipartimento di Matematica, Università di Torino, Torino, Italy

**Keywords:** shear stress in viral capsids, capsid structure, PRD1-adenovirus lineage

## Abstract

Many larger and more complex viruses deviate from the capsid layouts predicted in the seminal Caspar–Klug theory of icosahedral viruses. Instead of being built from one type of capsid protein (CP), they code for multiple distinct structural proteins that either break the local symmetry of the CP building blocks (capsomers) in specific positions or exhibit auxiliary proteins that stabilize the capsid shell. We investigate here the hypothesis that this occurs as a response to mechanical stress. For this, we construct a coarse-grained model of a viral capsid, derived from the experimentally determined atomistic positions of the CPs, that represents the basic features of protein organization in the viral capsid as described in Caspar–Klug theory. We focus here on viruses in the PRD1-adenovirus lineage. For *T* = 28 viruses in this lineage, which have capsids formed from two distinct structural proteins, we show that the tangential shear stress in the viral capsid concentrates at the sites of local symmetry breaking. In the *T* = 21, 25 and 27 capsids, we show that stabilizing proteins decrease the tangential stress. These results suggest that mechanical properties can act as selective pressures on the evolution of capsid components, offsetting the coding cost imposed by the need for such additional protein components.

## 1. Introduction

Viral capsids are protein containers that encapsulate and thus protect the genomic material between rounds of infection. In the majority of cases, viral capsids are organized with icosahedral symmetry, and their architectures can be modelled in terms of the polyhedral models in the Caspar–Klug quasi-equivalence theory. Smaller viruses, which typically have capsids assembled from multiple copies of a single type of capsid protein (CP), are fairly well described by this theory. By contrast, for larger and more complex viruses, such as those in the PRD1-adenovirus lineage, multiple deviations from these models have emerged. In some viruses, the hexagonal sites are occupied by different compositions of distinct types of CPs, thus breaking the local symmetry of the hexameric positions in the surface lattice. In others, there are additional protein components in specific positions, whose existence and locations cannot be explained in the context of Caspar–Klug theory. Here we investigate our hypothesis that these deviations from Caspar–Klug theory can be related to the mechanical properties—specifically, the built-in stresses—of the capsid shell. The importance of residual stress in many functions of the viral capsid has already been demonstrated, for instance in [1]. In our work, using the Caspar–Klug models as a starting point, we compute the stress distribution across the capsid with reference to these models. We show that local symmetry breaking and the occurrence of additional protein components can be correlated with the mechanical properties and curvature of these capsid shells.

A number of studies of the mechanical properties of viral capsids have previously been performed, both theoretically and experimentally (see [2,3]). This includes all-atoms molecular dynamics simulations (e.g. [4]), continuum models based on shell theory (e.g. [5]) and coarse-grained models in which whole proteins, or groups thereof, are represented by rigid or elastic bodies [1,6–9]; this also includes models in which the discrete nature of the shell is taken into account by suitable triangulations of the surface [10,11]. As capsids are intrinsically discrete structures, the details of the tessellation as well as the local organization into monomers, dimers, trimers, pentamers or hexamers have an impact on the mechanical properties of the capsid. Hence, it seems appropriate to use either molecular dynamics or coarse-grained models, since continuum models cannot take such details of the CP organization into account.

Here we use the coarse-grained model by Zandi & Reguera [6], a simple scheme in which the hexamers and pentamers are represented by spheres interacting with their neighbours via a Lennard-Jones potential. This model is able to capture the arrangement of the capsid building blocks (capsomers) as described by Caspar–Klug theory, and has proven to be useful to investigate a number of general features of viral capsids, such as buckling depending on the shape [8] or resistance to cracking [6].

According to Caspar–Klug theory, the arrangement of the CPs follows the principle of quasi-equivalence: CPs must locally have similar environments and group as 12 pentamers and a variable number of hexamers. The capsid can therefore be represented as a surface with a close-packed tessellation of pentagonal and hexagonal building blocks. Note, however, that the actual mechanical and assembly units, the capsomers, need not be pentamers and hexamers, but can also be single proteins, dimers or trimers [12]. Pentamers must have fivefold symmetry, because they are located on the particle fivefold symmetry axes. By contrast, hexamers do not need to have local sixfold symmetry, but can occur in distinct conformations formed from smaller units, thus violating the principle of quasi-equivalence. We explore here the mechanical reasons that may account for such non-quasi-equivalent architectures.

**Figure 1.**
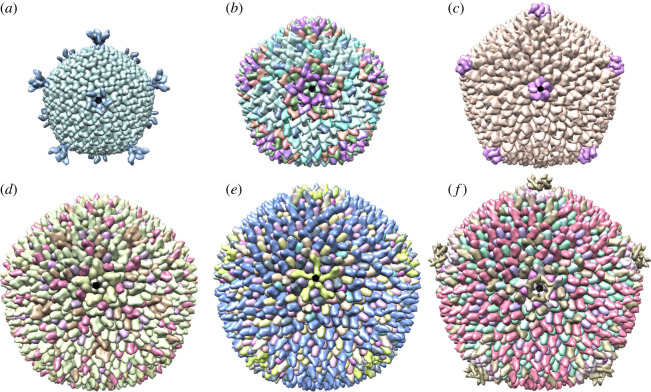
(*a*–*f*) Examples of viruses with non-quasi-equivalent architectures. (*a*) *Pseudoalteromonas virus* PM2, (*b*) *Pseudomonas virus* PRD1, (*c*) the *Mimivirus-dependent* Sputnik virus (PDB-ID: 2w0c, 1gw7, 3j26), (*d*) *Haloarcula hispanica* SH1 virus (SH1), (*e*) *Haloarcula hispanica* icosahedral virus (HHIV-2), (*f*) *Haloarcula californiae* icosahedral virus (HCIV-1) (PDB-ID: 6qt9, 6h82, 6h9c). PDB data for *Thermus* phage P23-77 are not available. All capsids are viewed along a fivefold axis.

In particular, we focus on the distribution of the residual shear stress in medium-sized capsids in the PRD1-adenovirus lineage (figure 1), spanning *T* = 21 to *T* = 28 architecture in size, because they exhibit a wide spectrum of different deviations from Caspar–Klug theory and are therefore ideal to test our hypothesis.

The capsids of these viruses fall into two classes: either they have a different organization of the major coat proteins at some of the hexameric positions at and around the twofold axes or they have ancillary cementing proteins that reinforce the shell, again near the twofold axes. Our analysis suggests that, in all viruses in the first class, the concentration of shear stress at these axes may be responsible for the local deviations from quasi-equivalence. For viruses in the second class, the situation is less definite, in that in some cases the loci of stress concentration do not coincide with the sites at which reinforcing proteins are located. This could be the result of us using coarse-grained models that are built from the atomic positions of the CPs, and thus they implicitly contain contributions from any auxiliary proteins at the inner capsid surface that are not captured by a simple model of the capsid shell. We therefore use a different strategy in this case. We compare the model derived from the biological data with a mathematical model of the capsid shell in isolation, showing that there is a significant stress reduction relative to ideal spherical or icosahedral mathematical rendering of the capsids that we are attributing to the presence of the auxiliary components. We also show that there is a correlation between the location of the reinforcing proteins and the sites at which curvature is concentrated, suggesting that these additional protein components may, at least in part, also be a response to curvature-related stresses. However, the latter is not dealt with explicitly here, as this is not possible in the context of our model due to the intrinsic limitations of Lennard-Jones interactions. Indeed, our model is appropriate to capture the local interactions between neighbouring capsomers when the curvature is small, and works better for capsids with small deviations from sphericity and where icosahedral edges are smooth. In summary, our analysis demonstrates that non-quasi-equivalent components in a complex viral capsid, in the form of either local symmetry breaking of the hexamers or the occurrence of additional protein components at the inner capsid shell, can be rationalized, at least in part, as a consequence of mechanical stress.

## 2. A coarse-grained capsid model

**Figure 2.**
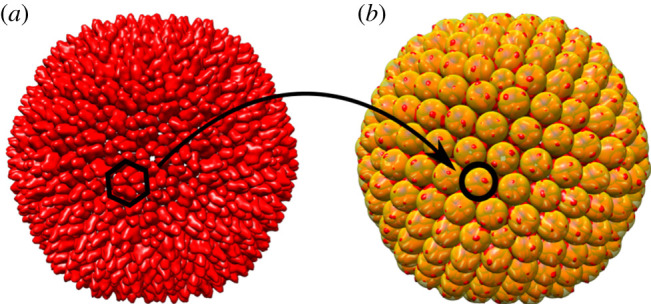
The coarse-graining procedure illustrated for the example of a *T* = 28 capsid. Hexamers in the PDB structure of the viral capsid (*a*) are replaced by spheres centred at the centres of mass of the atomic positions of each capsomer (*b*).

The coarse-grained model for spherical capsids introduced by Zandi & Reguera [6] is designed for capsids conforming to Caspar–Klug theory, for which pentamers and hexamers (capsomers in what follows) are the basic mechanical units. The capsid is idealized as a surface *S*, and the capsomers are represented as small spheres with centres on *S* (figure 2) that interact via Lennard-Jones forces. The total energy is given as the sum of all pairwise interaction potentials, and the stress is measured by the virial stress at zero temperature. All hexamers are modelled as indistinguishable and have the same size and mechanical properties, and the same holds for the pentamers.

In the original model of Zandi and Reguera (see also Aznar *et al.* [8]), the surface *S* is given either by a sphere or by a regular icosahedron. Indeed, a classical scaling argument [5] supports the idea that small capsids are nearly spherical, while large capsids are in a good approximation icosahedral. More refined theoretical arguments [13], however, suggest that capsids exhibit a larger variety of shapes, and are often multi-faceted or even slightly concave. The fact that many capsids of intermediate sizes do not fit well into the ‘sphere versus icosahedron’ dichotomy is also substantiated by the best-fit analysis of viral shapes in [8].

As using central force potentials in the presence of edges and corners would be inappropriate, we have based our analysis here on a point set obtained by computing the centroids of the actual hexamers and pentamers from experimental data (Protein Data Bank (PDB) files of the atomic positions in viral CPs), and have benchmarked our results against computations based on idealized spherical and icosahedral models. The calculations were performed with the software ‘UCSF Chimera’ [14] and MATLAB.

In our mathematical models of viral capsids, we denote by *X* = {***x***_*i*_}_*i*=1, …,*N*_ a capsid configuration that is defined as the set of points representing the centroids of the capsomers. We assume, without loss of generality, that the points are indexed so that the pentamers have indices *i* = 1, …, 12, and the hexamers *i* = 13, …, *N*. For the models based on the Zandi and Reguera approach, *X* ⊂ *S*, where *S* is either a sphere or an icosahedron, while for the structures obtained from the PDB data here we denote by *S* the triangulated surface whose nodes are the points in *X*.

The adjacency matrix of a configuration is defined as follows. For fixed *δ* > 0, we say that two points ***x***_*i*_ and ***x***_*j*_ are adjacent if their Euclidean distance is less than *δ*, and write

$$A_{ij}=\left\{ \begin{array}{ll} 1 & \text{if}\;|x_{i}-x_{j}|<\delta ,\\ 0 & \text{otherwise}. \end{array}\right.$$

The parameter *δ* is chosen to be of the order of the distance between the centres of two neighbouring capsomers, i.e. twice the radius of a typical hexamer. Note that a pentamer is adjacent to five hexamers, while a hexamer is adjacent to six capsomers (pentamers or hexamers).

We follow [6] and approximate the interaction between two capsomers indexed by *i*, *j* by a Lennard-Jones potential of the form

2.1 $$\begin{array}{ll} & V_{ij}(x_{i},x_{j})=V_{ij}(r_{ij})=\epsilon_{0}\left({\left(\frac{\sigma_{ij}}{r_{ij}}\right)}^{12}-2{\left(\frac{\sigma_{ij}}{r_{ij}}\right)}^{6}\right)\\ & \;r_{ij}=|x_{i}-x_{j}| \end{array}\}$$

Here, *σ*_*ij*_ denotes the equilibrium distance between the centres of the capsomers (see table 2 in appendix A.1), and *ε*_0_ a positive constant.

Departing slightly from [6,8], we write the total energy of the system as

2.2 $$E(X)=\sum_{i,j=1}^{N}A_{ij}V_{ij}(r_{ij}),$$

and, as a measure of the interaction forces at equilibrium, we take the static part of the local virial stress tensor at point ***x*** _*i*_ (e.g. [6,8] and formula (A.27) in [15]):

2.3 $$T_{i}(X)=\frac{1}{2|S|}\sum_{\;j\neq i}f_{ij}⊗r_{ij},\;r_{ij}=x_{j}-x_{i},$$

where

2.4 $$f_{ij}=-\frac{1}{r_{ij}}\frac{\text{d}V_{ij}}{\text{d}r}(r_{ij})A_{ij}r_{ij}$$

is the interaction force between capsomers *i* and *j* and |*S*| is the area of the capsid surface. The virial stress (here in its static version) is a common tool in the study of many-particle systems in molecular dynamics simulations. It is the analogue of the Cauchy stress for discrete media. As such, it is a measure of the interaction force between contiguous portions of a material across their common boundary. In our context, we can interpret it as a tool to study how the interaction forces tend to deform the bonds between the hexamers. This interpretation is discussed in some detail in the following section.

The forces {***f***_*ij*_}_*j*=1, …,*N*_ are internal to the capsid, being the interaction forces from all adjacent capsomers *j* ≠ *i* on capsomer *i*, and, in general, are not balanced, i.e. $\sum_{j}f_{ij}\neq 0$. In the equilibrium configuration of the capsid, as captured by the PDB file of the experimentally determined coordinates, these forces are balanced by the external forces (such as electrostatic interactions between the capsomers and the genomic material or membrane proteins, osmotic pressure or steric forces due to the confinement of the genome inside the capsid). Only the internal forces, however, contribute to the virial (or Cauchy) stress, which, by its very definition, is a measure of the contact forces internal to a material body.

### 2.1. Maximum tangential shear stress

**Figure 3.**
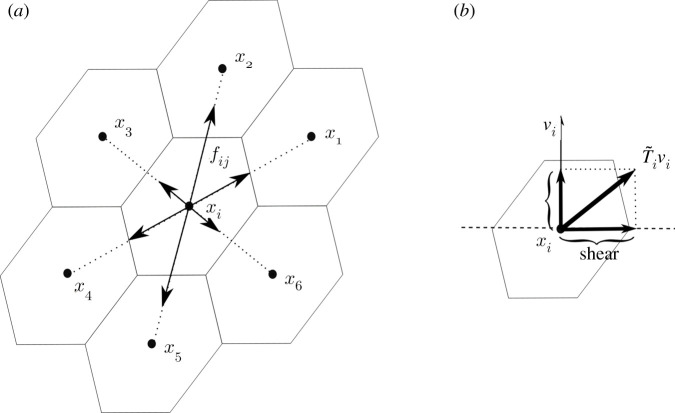
Schematics describing the relation between the forces acting on a hexamer, the virial stress and the shear stress in a planar arrangement of the deformed hexamers. (*a*) Interaction forces acting on the centroid ***x*** _*i*_ of the central hexamer *i* due to the surrounding hexamers. In this example, the forces are balanced, i.e. they add to zero. (*b*) Given a separating curve at ***x ***_*i*_ with unit normal ***ν***_*i*_ in the tangent plane to the capsid, ${\tilde{T}}_{i}\nu_{i}$ is the traction across the curve and the shear stress corresponds to the component of the traction tangential to the curve.

The stress ***T***_*i*_ can be decomposed into a part ${\tilde{T}}_{i}$ that is tangential to the surface *S* and a part that is normal to it, as discussed in appendix A.2. The stress tensor measures the contact interactions between different portions of a body across their common boundary. Here, the role of the body is played by the two-dimensional surface *S* as follows. Consider a curve dividing two portions of *S*, and denote by ***ν***_*i*_ the unit normal to this curve in the tangent plane to the surface at ***x***_*i*_. As sketched in figure 3, ${\tilde{T}}_{i}\nu_{i}$ is the traction across the curve. The shear stress is the component of this traction tangential to the curve and, intuitively, is the response to the sliding of the two portions of the surface relative to each other. The component of ${\tilde{T}}_{i}\nu_{i}$ normal to the curve is the tension along the direction ***ν***_*i*_. Clearly, both components depend on the direction along which they are computed.

If we label the centroids, as in figure 3, that correspond to the capsomers surrounding ***x***_*i*_ counter-clockwise as *j* = 1, …, 6 and assume that the forces are balanced with ***f***_*ij*_ = −***f***_*i*,*j*+3_ and ***r***_*ij*_ = −***r***_*i*,*j*+3_ for *j* = 1, 2, 3, then we obtain

$$T_{i}(X)=\frac{1}{2|S|}\sum_{\;j\neq i}f_{ij}⊗r_{ij}=\frac{1}{\left|S\right|}\sum_{\;j=1}^{3}f_{ij}⊗r_{ij}\neq 0.$$

Note that this holds even though $\sum_{\;j\neq i}f_{ij}=0$.

Indeed, ${\tilde{T}}_{i}$ is a symmetric tensor, and we denote its eigenvalues by *λ*_max,*i*_, *λ*_min,*i*_. These are the so-called principal stresses at ***x***_*i*_ and they correspond to the maximal and minimal tension along all possible directions ***ν***_*i*_. The lateral stress Λ_*i*_ at point ***x***_*i*_ is defined as the mean tension,

2.5 $$\Lambda_{i}=\frac{1}{2}(\lambda_{\max,i}+\lambda_{\min,i}),$$

and the maximum tangential shear stress at point ***x***_*i*_ is given by

2.6 $$\tau_{\max,\;i}=\frac{1}{2}(\lambda_{\max,\;i}-\lambda_{\min,\;i}),$$

which is attained along the direction forming a *π*/4 angle with the eigenvectors of ${\tilde{T}}_{i}$. More explicit representations of the lateral and maximum shear stresses are derived in appendix A.3.

### 2.2. Shape analysis and curvatures of the capsid

Lennard-Jones interactions are not designed to penalize curvature and, as such, our approach is not appropriate for the study of the stress distributions in capsids with sharp edges, such as perfectly icosahedral shells. Hence, in order to validate our results, it is useful to analyse the curvature of the capsids we discuss here. For a surface the curvature is measured by the so-called second fundamental form, whose invariants are the Gaussian and mean curvatures. In broader terms, the Gaussian curvature at a point measures how ‘peaky’ a surface is at that point, whilst the mean curvature can be viewed as a measure of the bending of the surface.

**Figure 4.**
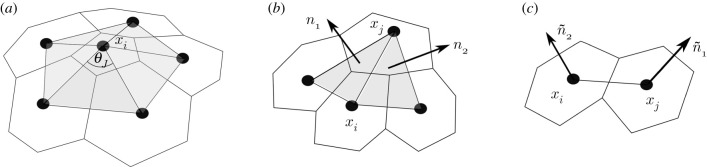
Illustration of the differences between the three discrete curvature measures used for the shape analysis: (*a*) Gaussian curvature, (*b*) mean curvature and (*c*) true curvature.

The advantage of using these curvature measures is that they can be generalized to triangulated surfaces, which is the case here. We use the definitions of [16]: the Gaussian curvature for a triangulated surface is a function that associates to each node *i* of the triangulation (here the centroids of the capsomers) the number

$$K_{i}=2\pi -\sum_{J}\theta_{J},$$

where *J* label the triangles with a vertex in *i* (*J* = 1, …, 6 and *J* = 1, …, 5 for *i* a hexamer and a pentamer, respectively) and *θ*_*J*_ are the internal angles at *i* of these triangles (figure 4*a*).

The mean curvature of a triangulated surface is a function defined on the edges of the triangulation, and is given in terms of the angle between the normals of two triangles that meet at that edge,

$$H_{ij}=2r_{ij}\sin\frac{\theta_{ij}}{2},$$

where *i* and *j* are adjacent vertices and *θ*_*ij*_ is the exterior dihedral angle along the edge, defined by cos *θ*_*ij*_ = ***n***_1_ · ***n***_2_. Here, ***n***_1_ and ***n***_2_ denote the unit normals to the triangles, pointing outward from *S* (figure 4*b*). For an icosahedron, the Gaussian curvature is concentrated at the vertices and the mean curvature at the edges, while for a sphere both curvatures are constant.

In this paper, we also use a different notion of discrete curvature that measures the angle between the capsomers. It is an analogue to the mean curvature, in that it is a function that assigns to each edge a measure of the angle between the capsomers (hexamers and pentamers) that meet at that edge. We define

$${\tilde{H}}_{ij}=2r_{ij}\sin\frac{\;{\tilde{\theta }}_{ij}}{2},$$

where *i* and *j* denote adjacent vertices as before and $\;{\tilde{\theta }}_{ij}$ is defined by $\cos\;{\tilde{\theta }}_{ij}={\tilde{n}}_{1}\cdot {\tilde{n}}_{2}$. Here, ${\tilde{n}}_{1}$ and ${\tilde{n}}_{2}$ denote the unit normals to the capsomers pointing outward from *S* (figure 4*c*).

We shall use this curvature measure only for the discrete surfaces derived from the PDB data, since the software Chimera allows us to compute the orientation of the median planes of the capsomers. For the surfaces generated by the Zandi–Reguera procedure, there is no information about the orientation of the capsomers, and the true curvature is therefore not defined.

## 3. Applications to non-quasi-equivalent viral architectures in the PRD1-adenovirus lineage

In order to determine the influence of the tangential shear stress on the structural features of the capsid, we perform a case study of viruses in the PRD1-adenovirus lineage because they cover capsid architectures that deviate from the quasi-equivalence principle in Caspar–Klug theory in different ways. Viruses in this lineage infect organisms from all three domains of life and exhibit similar structural features, such as a common capsid architecture and coat protein folds [17]. These viruses either have two major CPs, whose arrangement violates the Caspar–Klug paradigm in a number of hexamers, as is the case for viruses with *T* = 28 capsids, or they exhibit cementing and other minor stabilizing structural proteins (as in the *T* = 21, *T* = 25 and *T* = 27 capsids) whose locations at special positions in the capsid cannot be explained via Caspar–Klug theory. We address here the hypothesis that the non-uniform structure of the capsid could be a means of accommodating the excess residual stress at special locations, and thus constitute an evolutionary response to stress concentrations at specific sites in the capsid shell.

### 3.1. Non-quasi-equivalent hexamers in *T* = 28 capsid architectures

**Figure 5.**
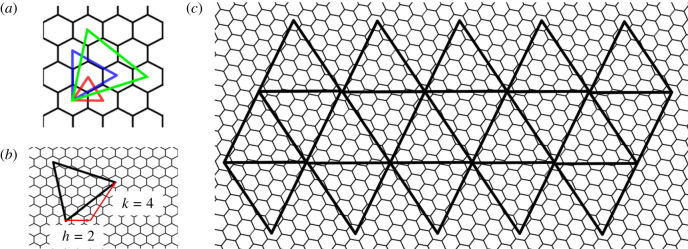
Caspar–Klug models of virus architecture. The *T*-number identifies different ways of superimposing an equilateral triangle on a hexagonal grid; (*a*) the examples *T* = 1 (*h* = 1, *k* = 0; red), *T* = 3 (*h* = 1, *k* = 1; blue) and *T* = 4 (*h* = 2, *k* = 1; green) are shown. (*b*) Each triangle is defined by its edge length, which is characterized by steps between hexagonal midpoints along two lattice directions *h* and *k* at a counterclockwise *π*/3 angle; the case (*h*, *k*) = (2, 4), corresponding to a *T* = 28 *dextro* capsid, is shown. (*c*) Twenty such triangles define an icosahedral surface, and its embedding into a hexagonal grid shows the organization of hexagonal faces, each representing six proteins in the viral surface lattice of the *T* = 28 capsids.

We first consider capsids formed from two different types of major CPs that exhibit distinct types of hexamers in the capsid surface. We direct our investigations to *Haloarcula hispanica* SH1 virus (SH1), *Thermus* phage P23-77, *Haloarcula hispanica* icosahedral virus (HHIV-2) and *Haloarcula californiae* icosahedral virus (HCIV-1); see figure 1*d*–*f*. The overall organization of these capsids can be described in terms of Caspar–Klug theory (figure 5*b*,*c*). According to the quasi-equivalence paradigm of Caspar & Klug [18], every protein in the capsid has approximately the same environment, and the geometric structure of a virus can be modelled by superimposing the planar layout of an icosahedral surface onto a close-packed hexagonal tessellation made of repeated copies of a single protein. The way in which the icosahedron is superimposed onto the planar tessellation determines the so-called *T*-number, which is defined as *T* = *h*^2^ + *hk* + *k*^2^, where *h* and *k* are positive integers, one possibly being zero. This defines a planar embedding of an icosahedral surface into a hexagonal lattice as illustrated in figure 5*b*,*c*. *T* corresponds to the number of proteins in the fundamental domain of the representation of the icosahedral group in this construction.

**Figure 6.**
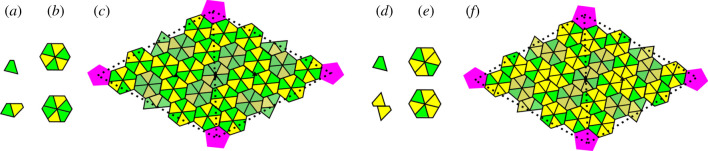
Maps outlining the protein positions in the *T* = 28 capsids in the PRD1-adenovirus lineage. (*a*–*c*) SH1, HCIV-1 and HHIV-2. VP4 and VP7 correspond to tiles coloured in yellow and green, respectively. (*a*) The capsid building blocks: (*top*) a VP7 monomer; (*bottom*) a VP4-VP7 heterodimer. (*b*) (*top*) A capsomer bearing three turrets; (*bottom*) a capsomer with only two turrets. (*c*) The two distinct local protein configurations are shown on two icosahedral faces. The hexamers with two turrets (shaded hexagons) are located adjacent to (and at) the twofold axes. A twofold axis is indicated by a black ellipse, and threefold axes by black triangles. (*d*–*f*) P23-77. VP16 and VP17 are coloured in yellow and green, respectively. (*d*) The capsid building blocks: (*top*) a VP17 monomer; (*bottom*) a VP16 homodimer. (*e*) The two different types of hexamers. (*f*) Two icosahedral faces: the three hexamers adjacent to, and at, the twofold axes are shaded.

The Caspar–Klug scheme, however, does not fully explain the structures of the capsids studied in this section, as the hexameric positions are occupied in distinct ways by two different types of proteins that form dimers and monomers, and are therefore not quasi-equivalent. In particular, the capsids of SH1, HHIV-2, HCIV-1 and P23-77 have a pseudo *T* = 28 *dextro* surface lattice. However, the two types of CP break the local symmetry of the hexamers. There are two distinct ways in which this occurs (figure 6): the two types of CP form a heterodimer and one of the CP types also occurs as a monomer; or, one type of CP occurs as a homodimer and the other one as a monomer. These are discussed as scenario 1 and scenario 2 below.

**Figure 7.**
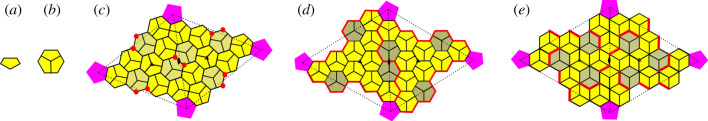
Surface architectures of PM2, PRD1 and Sputnik. (*a*) Schematic of the subunit given by the major CP. (*b*) A sketch of the hexamer formed from three copies of the major CP. (*c*) PM2: the locations of hexamers with respect to two icosahedral faces. Red dots denote the sites at which the cementing proteins P6 are anchored to the lipid membrane underneath the capsid. The hexamers adjacent to the twofold axes are indicated by shading, and pentons coloured in magenta as before. (*d*) PRD1: positions of the hexamers with respect to two icosahedral faces. The red lines indicate the locations of the tape-measure proteins that reinforce the capsid. The hexamers adjacent to the twofold axes are shaded. (*e*) Sputnik: positions of the hexameric units with reference to two icosahedral faces. Red lines denote the sites at which the cementing proteins are anchored to the CPs. Three hexamers in symmetric positions around the threefold axes are shaded.

*Scenario 1* (figure 6*c*). In HHIV-2, HCIV-1 and SH1, the major CPs VP4 and VP7 form dimers (heterodimers in what follows, because they are made of two different proteins; figure 6*a*), which in turn combine to form pseudo-hexameric capsomers with three or two towers [19]. All three viruses have the same protein organization within the icosahedral fundamental domain, consisting of a copy of a penton protein at the fivefold axis (magenta), 12 copies of VP4 (yellow) and 15 copies of VP7 (green). The two types of hexamers, one with two towers and the other with three (called *type II* and *type III* hexamers), are formed from heterodimers and monomers as illustrated in figure 6*b*. Three-tower hexamers are built from three copies of the heterodimer VP7–VP4, while the two-tower hexamers are made by two heterodimers and two unpaired VP7 subunits (VP4 bears the tower, while VP7 has none). Hexameric units are non-quasi-equivalent, because they can have two different types of organizations (whilst, by contrast, all hexamers in a classical Caspar–Klug capsid must be indistinguishable). The 90 two-towered capsomers sit in special symmetric positions at the twofold axes (shaded capsomers in figure 6*c*). Note that the type II hexamers are adjacent to and located at the twofold axes and their structures are distinct from those of the other hexamers. Type III hexamers stick together by two strong peg-in-hole interactions at each hexamer interface, while type II hexamers have many fewer interactions. They are therefore perhaps better suited to absorbing the build up of shear stress, thus explaining their occurrence in specific positions in the hexagonal surface lattice. We will show below that these hexamers indeed are located at hot spots of tangential shear stress (red/orange in figure 7*a*–*c*).

*Scenario 2* (figure 6*f*). The major CPs of HHIV-2 and HCIV-1 are structurally similar, regardless of their scarce sequence similarity, to the ones of *Thermus* phage P23-77 and the individual *β*-barrels of the double *β*-barrel major CP in marine bacteriophage PM2, which is considered to be the most ancient member of the PRD1-adenovirus lineage.

We use P23-77 in order to illustrate the second layout according to which CPs can be organized in the capsid shell. The P23-77 capsid is made from pseudo-hexameric units formed from two major CPs, VP16 and VP17, that give the shell a typical crenelated appearance [20]. VP16 and VP17 have a high structural homology to the major CPs in HHIV-2: in particular, VP17 contains two domains that sit atop each other, as in VP4, while the small VP16 contains just one domain. VP16 does not exist as a single protein in the capsid, but its native state is that of a dimer of intertwined sub-units. All the VP16–VP16 homodimers in the capsid sit across the boundaries of the hexameric units. These display four copies of VP16 (yellow) and two of VP17 (green) that attach to one VP16 in the hexamer via a specific site (figure 6*d*). Since the dimer does not have a turret domain, in contrast to VP17, all the hexameric units bear two turrets each. However, their arrangement is different in the hexamers in special symmetric positions at the icosahedral twofold axes, in that the hexamers have twofold rather than a lack of rotational symmetry (figure 6*e*).

Note that the major CPs of P23-77 have structural similarities to those of SH1 in scenario 1 above, but the arrangement of the turrets differs between the two capsids [21]. In fact, SH1, besides having two-tower capsomers, also has capsomers with three turrets, giving the shell a crenellation different from that of P23-77. Even though the protein organization within individual hexamers is different from scenario 1, the capsid of P23-77 also has the second hexamer type at the positions where the tangential shear stress concentrates (cf. figures 6*f* and 7*d*). Indeed, both capsid architectures exhibit two types of hexameric protein clusters, with, as we shall see, one type located at the positions of maximal shear stress.

### 3.2. Capsid architectures with auxiliary proteins

We next consider those members of the lineage for which the arrangement of the major CPs satisfies the quasi-equivalence principle, but for which minor or cementing proteins are present that are not explained by the Caspar–Klug scheme.

**Figure 8.**
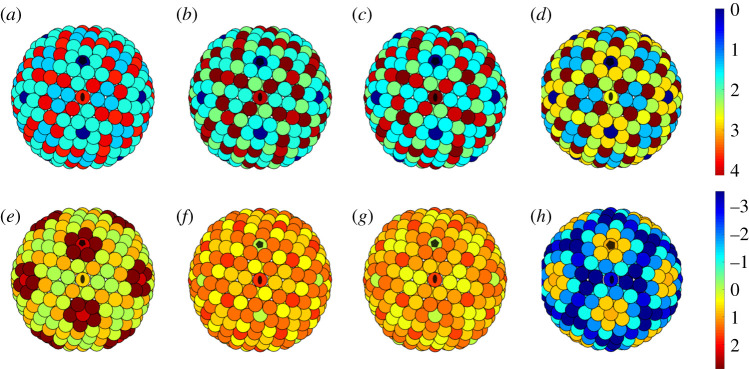
(*a*–*d*) Distribution of the maximum tangential shear stress for the *T* = 28 capsids of (*a*) SH1, (*b*) HCIV-1, (*c*) HHIV-2, (*d*) P23-77 viewed along a twofold axis. The concentration of shear stress at the three hexamers at, and near, the twofold axes in (*a*–*c*) is apparent (cf. figure 6). Recall that the capsid in (*d*) is given by the distribution of points on a sphere instead of the actual PDB file. (*e*–*h*) Lateral stress for the *T* = 28 capsids of (*e*) SH1, (*f*) HCIV-1, (*g*) HHIV-2, (*h*) P23-77 viewed along a twofold axis. Black ellipses and pentagons denote twofold and fivefold axes, respectively. Notice that the maximum value of the shear stress is almost twice the maximum value of the lateral stress (stress units are arbitrary).

The first example we consider is the marine lipid-containing bacteriophage PM2 (figure 1*a*), which is an icosahedral pseudo *T* = 21 virus in the PRD1-adenovirus lineage [22]. The major CP P2 forms the capsomer, which corresponds to three copies of interlocking subunits, each of them displaying a double *β*-barrel fold. This organization gives the hexamer pseudo-sixfold symmetry (figure 8*a*,*b*).

Protein P1 contributes to the pentons at the fivefold axes, while P3 to P10 are membrane-associated proteins connecting the capsid to the lipid bilayer that encloses the genome. In particular, the arrangement of the proteins P6 and P3 obeys icosahedral symmetry and could help stabilize the capsid. P6 is located adjacent to the two hexamers closest to the twofold axes (figure 8*c*).

As a second example, we consider PRD1 (figure 1*b*), which gives the name to the lineage. PRD1 is a bacteriophage containing a membrane that encapsulates a double-stranded DNA. The major CP P3 coincides with the hexamers and arranges in a pseudo-*T* = 25 shell, whereas protein P31 forms the pentamers at the fivefold axes (figure 8*d*). The remarkable feature of this capsid is the presence of 60 copies of a so-called tape-measure protein (P30), which extends from the pentons along the edge of the facets towards the twofold axes as shown in figure 8*d*, thus stabilizing the capsid [23,24].

The third example is the pseudo *T* = 27 capsid of the Sputnik virophage (1*c*), which is formed from 12 pentons sitting at the icosahedral fivefold axes and by several pseudo-hexameric capsomers, displaying three copies of a double *β*-barrel monomer as in the previous examples.

Here the multiple copies of a minor CP are located at the boundaries of the hexamers [25], as shown in figure 8*e*.

## 4. Results

Structural features that cannot be explained with Caspar and Klug’s quasi-equivalence theory present themselves in two principally different ways: either via symmetry breaking within hexamers or via additional structural proteins that stabilize the capsid. These two distinct types of exceptions to quasi-equivalence theory require a different interpretation of our results.

In §4.1, we focus on capsids made of two different major CPs, such as those described in §3.1. In these cases, hexamers have similar overall shapes but distinct internal compositions owing to the different ways in which monomers are arranged. As the Lennard-Jones model cannot account for the internal structures of the hexamers, our computed stress distribution only captures the arrangement of the hexamers in the capsid, and not their different material responses. However, since the two types of hexamers have approximately the same shape, the stress distribution we compute—notwithstanding the simplicity of the model—is a reasonable approximation of the actual stress distribution in the capsid due to the overall geometry (in terms of the *T*-number of the capsid). The fact that the hexamer structure is different exactly at those points where there is a high stress concentration suggests that this different internal structure is a response to the need of the hexamers to accommodate the excess stress, and points to a strong correlation between structure and residual stress.

In §4.2, we consider capsids in which all hexamers have the same structure, thus strictly following Caspar–Klug’s quasi-equivalence theory in the capsid, but in which additional reinforcing proteins, such as those introduced in §3.2, occur that are not explained by Caspar–Klug theory. However, since the computed stress distribution is a function of the geometry of the capsid, it already takes into account the modification of the capsid geometry due to the effect of the reinforcing proteins. Therefore, concentration effects in this distribution cannot be used to argue that these are a response to Lennard-Jones forces. Indeed, care has to be taken when interpreting the results, and we adopt the following strategy: as experimental data for the capsid in the absence of the reinforcing proteins are not available, we compare the stress distribution computed based on the structural data, i.e. in the actual capsids, with spherical and icosahedral models of capsids. A comparison reveals that there is a stress reduction in the actual structures, allowing us to conclude indirectly that the auxiliary structural proteins result in stress reduction in the capsid shell.

### 4.1. Non-quasi-equivalent hexamer positions in response to shear stress

In this subsection we focus on the *T* = 28 capsids of SH1, HCIV-1, HHIV-2 and P23-77; see §3.1. In order to support our conjecture that the internal structure of the hexamers is a response to the concentration of the shear stress, we computed the maximum shear stress and the lateral stress for the capsid configurations obtained from the PDB files, as illustrated in figure 7. Since, for the P23-77 capsid, there is no PDB file available in the literature, our computations for that capsid were instead based on the spherical codes with icosahedral symmetry of Hardin *et al*. [26]. According to Aznar *et al*. [8], the best choice among the spherical codes is given by the arrangement of points on a sphere that maximizes the volume of their convex hull, since these point sets [26] approximately minimize the Lennard-Jones energy. This assertion has been verified numerically in [8] via Monte Carlo methods, and we have also independently tested it. The high sphericity of the P23-77 capsid is indeed implied in table C1 of Aznar *et al.* [8] (form factor 0.61) and confirmed in, among others, [27].

Note, moreover, that all *T* = 28 capsids studied here exhibit a high degree of sphericity. This is supported by the curvature analysis in figure 13: the Gaussian curvature concentrates at the fivefold vertices that protrude from the capsid, the faces are not flat and the mean curvature does not strongly concentrate at the icosahedral edges, as would be the case in perfectly icosahedral capsids (figure 14). This validates our approach based on Lennard-Jones forces. In spherical surfaces, the curvature effects are smoothed out over the whole surface, so that Lennard-Jones interactions are appropriate to capture the nearest-neighbour interactions between the capsomers that involve compression or extension tangential to the surface.

Our first main result is that the values of the shear stress are consistently much larger than the values of the lateral stress, as shown in table 1. This confirms that the tangential shear stress should be more important than the lateral stress for the structure and mechanical properties of the capsid. Hence, it is reasonable to investigate the distribution of the tangential shear stress in relation to the positions of capsid features violating quasi-equivalence. The plots in figure 7 show the stress distributions, where the colour code indicates the magnitude of the stress. In all cases, the maximum shear stress concentrates at the hexamers with a different internal structure, suggesting that such deviations from quasi-equivalence might occur as a means of counteracting shear stress.

**Table 1.** Comparison between the maximum shear stress and the maximum lateral stress for the *T* = 28 capsids. The maxima are computed over all capsomers.

	SH1	HCIV-1	HHIV-2	P23-77
lateral stress: max _*i*_ |Λ_*i*_ |	2.9013	1.7044	1.6271	3.5817
shear stress: max_*i*_(*τ*_max,*i*_)	3.8703	4.3264	4.3217	4.3881

### 4.2. Auxiliary proteins breaking icosahedral capsid symmetry

**Figure 9.**
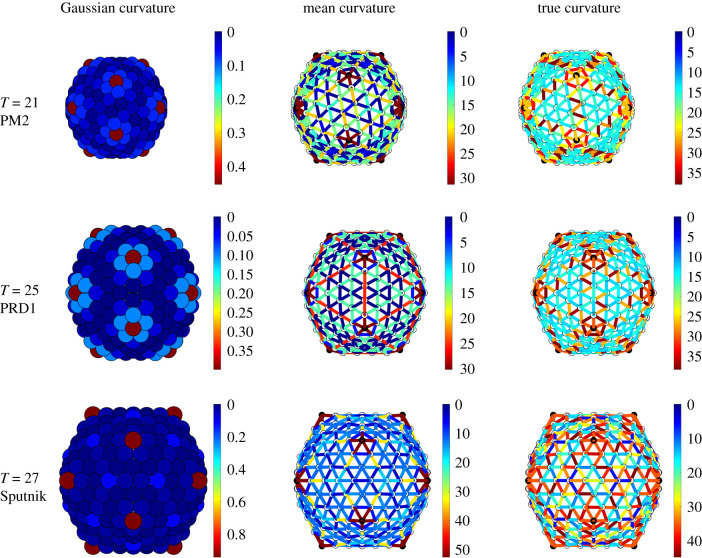
Gaussian, mean and true curvatures of the capsids of PM2, PRD1 and Sputnik. Notice the concentration of mean and true curvature at the icosahedral edges of PRD1 and PM2, respectively. The Gaussian curvature peaks at the fivefold axes, as expected. Notice that the icosahedral faces are almost flat (dark blue) only for PRD1. The distribution of curvature of the Sputnik is compatible with the shape of a pentakis-dodecahedron. The Gaussian curvature is measured in radiants, while the mean and true curvature are measured in angstrom.

In this subsection, we focus on the *T* = 21, 25 and 27 capsids of PM2, PRD1 and Sputnik; see §3.2. For these capsid architectures, the correlation between the stress and the deviation from quasi-equivalence is much weaker than for the *T* = 28 capsids. We therefore investigate in these cases whether the positions of the additional structural proteins correlate with bending. We first focus on the *T* = 21 capsid of PM2. The curvature plots (figure 9—top row) reveal that the Gaussian curvature concentrates at the fivefold axes, as expected, while the mean curvature is indeed larger at the icosahedral edges than at the faces, even though it does not fully vanish there. This means that the actual capsid shape is neither spherical nor icosahedral, but an interpolant between these extremal options. Interestingly, the true curvature concentrates more strongly than the mean curvature at the icosahedral edges, while it is uniform on the icosahedral faces. Since the true curvature measures how much two neighbouring hexamers are bent relative to each other, this suggests that the cementing proteins sitting at the twofold axes play the role of reinforcing the hexamer–hexamer attachment at sites where they tend to be strongly bent.

**Figure 10.**
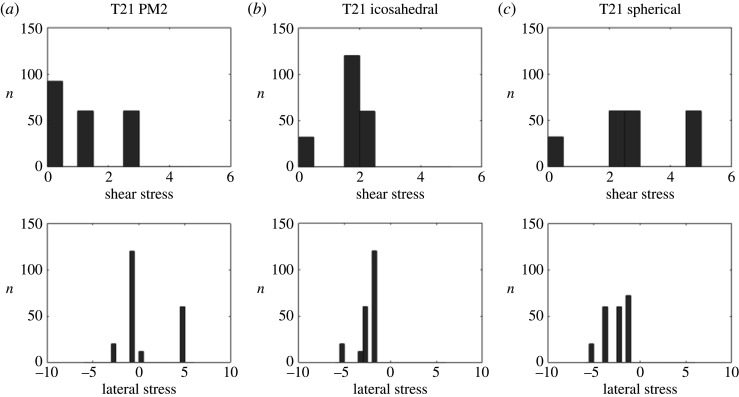
Histograms of the stress distribution for the *T* = 21 capsid, showing the number *n* of capsomers in each interval of stress values (in arbitrary units). Note that the actual capsid (*a*) exhibits a lower stress concentration than the corresponding icosahedral (*b*) and spherical (*c*) shapes.

The analysis of the stress distributions in figure 15 does not show a significant stress concentration at the twofold axes. However, there is a clear reduction in the overall stress relative to the idealized icosahedral and spherical models (figure 10). The histogram representation of the stress shows that the actual capsid structure of PM2 has fewer or no sites at which the stresses take extreme values, since the tails of the stress distribution are shorter for PM2 than for the icosahedral and spherical structures. This indicates that stress reduction may be an important determinant of capsid shape.

**Figure 11.**
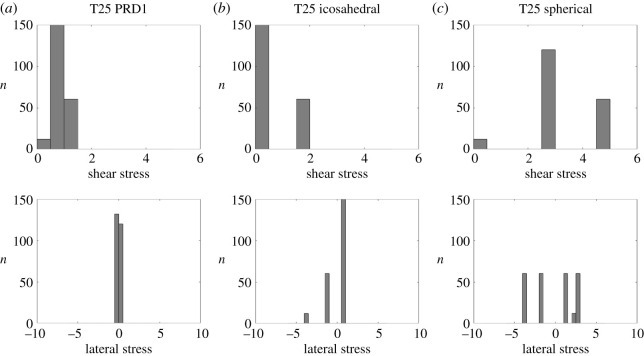
Histograms of the stress distribution for the *T* = 25 capsid, showing the number *n* of capsomers in each interval of stress values (in arbitrary units). Note that the actual capsid (*a*) exhibits a lower stress concentration than the corresponding icosahedral (*b*) and spherical (*c*) shapes.

For the *T* = 25 PRD1 structure, the mean curvature concentrates at the icosahedral edges, suggesting that the shape is close to icosahedral (figure 9, middle row). However, both the Gaussian and the mean curvatures are invariant under the full (120 elements, also containing reflections) icosahedral symmetry, as in a conventional *T* = 25 Caspar–Klug structure. The true curvature, however, is not reflection-invariant. The hexamers bend, following the same pattern as the tape-measure protein that is located at the inner capsid surface. Figure 8*d* suggests that the positions of the tape-measure proteins may be a response to curvature stresses. In addition, the shear stress concentrates at the twofold axes, again suggesting a possibly strong correlation with the positions of the tape-measure proteins. The latter could therefore play a role in reinforcing the capsid in the regions that must support higher stresses. Further, we observe a substantial stress reduction in the actual structure relative to the icosahedral and spherical capsids (figure 16), as well as an overall reduced stress concentration (figure 11).

**Figure 12.**
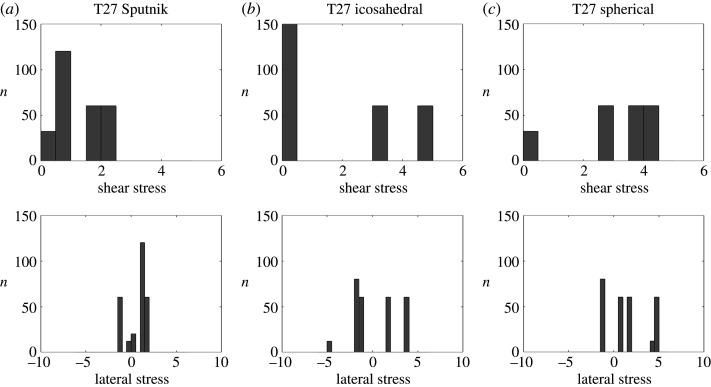
Histograms of the stress distribution for the *T* = 27 capsid, showing the number *n* of capsomers in each interval of stress values (in arbitrary units). Note that the actual capsid (*a*) exhibits a lower stress concentration than the corresponding icosahedral (*b*) and spherical (*c*) shapes.

Finally, in the *T* = 27 capsid of Sputnik, there is a substantial concentration of the true curvature along three bands joining the threefold axes to the fivefold axes. These appear to correlate with the positions of the cementing proteins (figures 9, bottom row and 8*e*). Hence, the same considerations as before also apply here. Even though the distribution of the stresses does not directly correlate with the locations of the cementing protein (figure 17), we again observe the absence of strong loci of stress concentration in the model of the Sputnik capsid derived from the PDB data, in contrast to the idealized icosahedral and spherical models (figure 12). As before, this suggests that the presence of the cementing proteins, whose effect on capsid architecture is taken into account implicitly via the PDB data but is absent in the idealized models, makes a contribution to stress reduction in the capsid.

**Figure 13.**
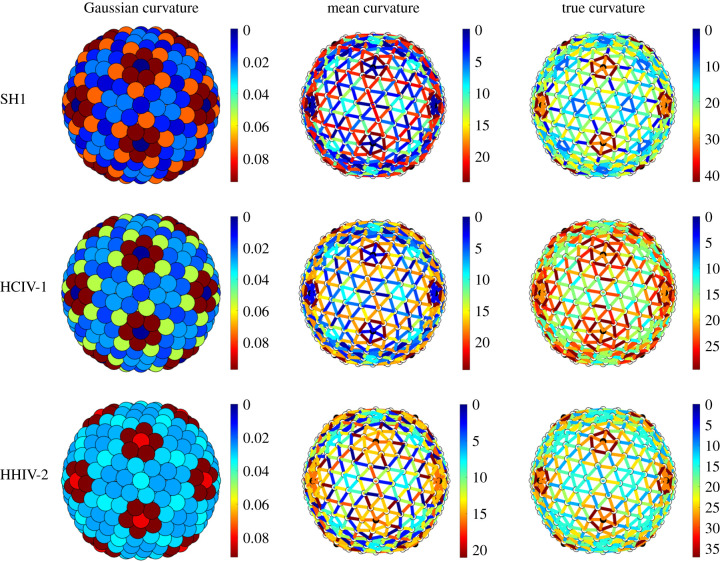
Gaussian, mean and true curvatures of *T* = 28 capsids. The lack of flat faces in comparison with the curvatures of the idealized spherical and icosahedral structures in figure 14 suggests that the shape of the capsid is better approximated by a sphere than by an icosahedron.

**Figure 14.**
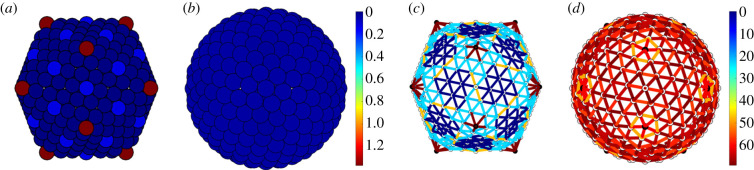
Comparison of icosahedral and spherical geometries for a *T* = 28 structure. Gaussian curvature for (*a*) icosahedron and (*b*) sphere; mean curvature for (*c*) icosahedron and (*d*) sphere (see also figure 13).

**Figure 15.**
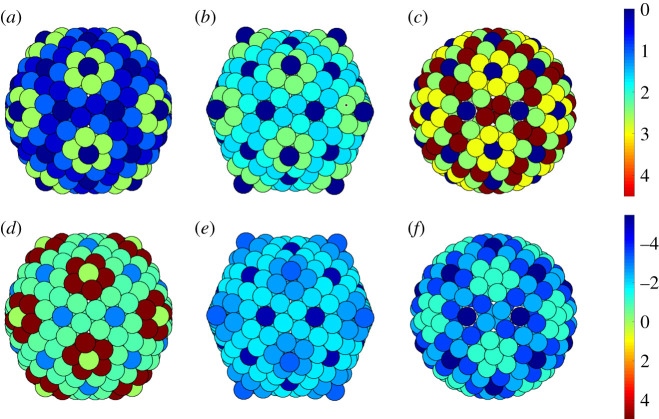
Stress distributions for *T* = 21 capsid architectures. (*a*–*c*) Distribution of the maximum shear stress for the PM2, icosahedral and spherical structures, respectively. (*d*–*f*) Distribution of the lateral stress for the same structures. Notice the reduction of the stress in the models based on actual capsid data relative to the purely theoretical structures.

**Figure 16.**
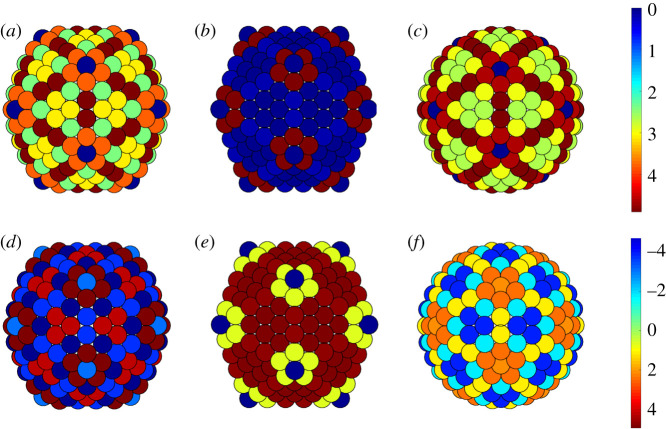
(*a*–*c*) Stress distributions for *T* = 25 capsid architectures: distribution of the maximum shear stress for the PRD1, icosahedral and spherical structures, respectively. (*d*–*f*) Distribution of the lateral stress for the same structures. Notice the reduction of the stress in the models based on actual capsid data relative to the purely theoretical structures, as well as the concentration of shear stress at twofold axes.

**Figure 17.**
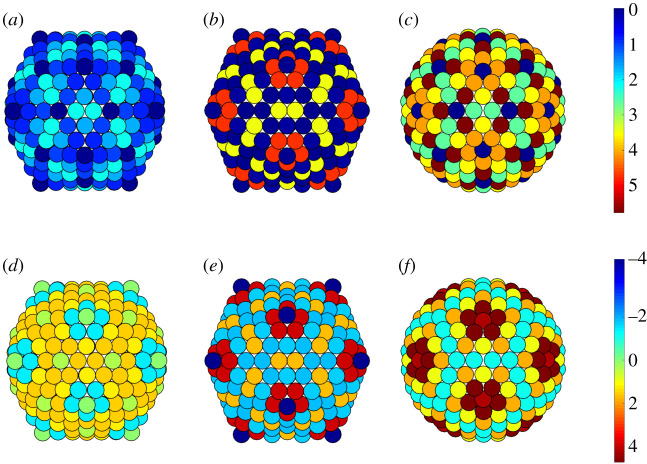
(*a*–*c*) Stress distributions for *T* = 27 capsid architectures: distribution of the maximum shear stress for the Sputnik, icosahedral and spherical structures, respectively. (*d*–*f*) Distribution of the lateral stress for the same structures. Notice again the reduction of the stress in the models based on the actual capsid data relative to the purely theoretical structures.

## 5. Discussion

Our model suggests a deep connection between the mechanical properties of viral capsids, in particular the distribution of the residual stresses, and the structural organization of the CPs, consistent with earlier studies [6,8]. Our analysis reveals that tangential shear stress is particularly important for the *T* = 28 viruses in the PRD1-adenovirus lineage, suggesting a possible explanation for the occurrence of different types of hexamers with distinct types of internal organization at specific locations in the capsid. In particular, the shear stress provides a possible explanation for the observed symmetry breaking and deviations from Caspar–Klug’s quasi-equivalence theory. Interestingly, the *T* = 28 viruses discussed here are the only known viruses displaying this pseudo *T*-number capsid architecture, suggesting that only viruses with distinct types of CPs can realize such capsid geometries, owing to shear stress.

For the other viruses in the same lineage, which are characterized by the occurrence of additional protein components stabilizing the capsid, there is a clear relation between the decrease in residual stress and the presence of these additional proteins. However, contrary to the *T* = 28 case, our model cannot be used directly to explain the location of the stabilizing proteins. This is because, in the PDB data of the atomic positions of the CPs that form the basis of our models, the impact of such components on capsid organization is implicitly contained. In this case, a comparison of our models derived from the experimental PDB data and the idealized models lacking such effects from additional components has enabled us to probe their impacts indirectly. This again revealed a possible role of these auxiliary structural proteins in stress relief.

Recently, a new classification scheme for virus structure has been introduced [28]. This models capsid architecture via a wider range of surface lattices, and contains the capsid geometries of Caspar–Klug theory as special cases. This extended scheme is in particular required for viruses with capsids formed from more than one type of CP. For example, this includes capsid architectures in which structural proteins occupy not only hexameric positions as in the examples discussed here, but there are also smaller CPs occupying trimeric positions. In herpes simplex virus 1 (HSV-1) [29], for example, these trimeric positions are occupied by heterotrimers, consisting of a dimer and monomer formed from different types of minor CPs. This local symmetry breaking within the heterotrimer is akin to that of the hexameric positions discussed here. It is therefore likely that the stress distribution across the capsid could also explain such heterotrimer arrangements. This would also be consistent with the fact that the hexamers in HSV-1 are identical and stabilized by another structural protein, and therefore cannot absorb the stress as in the examples discussed here. The proteins at the trimeric positions, on the other hand, would be able to do this.

In any case, mechanical stress provides a possible explanation for why such viruses code for more than one type of CP exhibiting heterogenic hexamers or, in the case of HSV-1, trimers, despite the additional demands on coding capacity. It appears that the need to relieve mechanical stress, in particular the concentration of tangential stresses, could be a driver for the evolution of such additional structural proteins, outweighing coding costs. Our analysis also begs questions regarding the evolution of viral capsid architectures over larger time scales. If mechanical stress is an evolutionary pressure impacting on the types and numbers of the CPs, as our study suggests, then it is perhaps not surprising to see only a limited number of different CP morphologies. Phylogenetic classification based on CP homology irrespective of the level of sequence homology, as proposed by Bamford *et al.* [30], therefore captures such essential evolutionary drivers.

## Data accessibility

This article has no additional data.

## Appendix A

**A.1. Equilibrium inter-capsomer distances**

The values for *σ*_*ij*_ are assigned as follows: for the model based on the PDB data, and *i*, *j* corresponding to distinct hexamers, these are the averages, over all hexamers, of the distances between the centroids of adjacent capsomers, i.e. $\sigma_{ij}:=\sigma_{hh}=(1/2M)\sum_{\;p,q>12}A_{\;pq}|x_{p}-x_{q}|$, where *M* = *N*(*N* − 1)/2 − 60 is the number of links between adjacent hexamers. For *i*, *j* corresponding to a hexamer and a pentamer, on the other hand, we compute the minimum distance between the pentamer centroid and the centroids of the adjacent hexamers, i.e. *σ*_*ij*_ := *σ*_*hp*_ = min_*p*≤12,*q*>12_|***x***_*p*_ − ***x***_*q*_|. For the spherical and icosahedral models, the capsomers are approximated by spheres with different radii according to whether they are pentamers or hexamers. Following [6], we assume that the equilibrium distances between the centres of two adjacent capsomers are the sum of the radii of the corresponding inscribed spheres. Taking the computed value of *σ*_*hh*_/2 for the radius of a hexamer, then *σ*_*ij*_ = *σ*_*hh*_ and *σ*_*ij*_ = (1 + *λ*)*σ*_*hh*_/2, for *i*, *j* both hexamers or a hexamer/pentamer pair, respectively, and *λ* = tan (*π*/6)/tan (*π*/5) (table 2).

**Table 2.** Inter-capsomer distances: *σ*_*hh*_ and *σ*_*hp*_ correspond to the average distances in Å between adjacent hexamers and pentamers, respectively.

	SH1	HCI V-1	HHIV-2	P23-77	PM2	PRD1	Sputnik
*σ*_*hh*_	85.4080	86.0775	85.9620	85.6209	72.9759	71.8629	76.1052
*σ*_*hp*_	72.3427	73.1553	74.0641	76.8300	63.4528	64.8080	77.1863

**A.2. Tangential projections**

When *S* is a sphere or an icosahedron, the unit normal to *S* is well defined away from the edges and corners. However, when *S* is a triangulated surface, the outward unit normal to *S*, which in turn defines the tangent plane to *S*, is only defined on the triangular faces. In that case, we choose a different approach: for each capsomer, we compute its habit plane from the PDB data and define the outward unit normal to *S* at the capsomer centroid as the unit normal to the habit plane of the capsomer.

In any case, denoting by ***n***_*i*_ a choice of the outward unit normal to *S* at point ***x***_*i*_, the tangential part of the stress is defined to be $\tilde{T}=P_{i}^{⊤}T_{i}P_{i}$, with ***P***_*i*_ = ***I*** − ***n***_*i*_ ⊗ ***n***_*i*_ the projection onto the plane orthogonal to ***n***_*i*_.

**A.3. An alternative expression for the lateral and maximum tangential shear stresses**

In order to obtain a more explicit representation of the maximum shear stress, fix a basis (***E***_*i*,1_, ***E***_*i*,2_) for the tangent plane to *S* at point ***x***_*i*_ and represent ${\tilde{T}}_{i}$ in this basis by the matrix

$$\left(\begin{array}{cc} \alpha_{i} & \beta_{i}\\ \beta_{i} & \gamma_{i} \end{array}\right),$$

with $\alpha_{i}={\left({\tilde{T}}_{i}\cdot E_{i,1}\right)}^{2}$, $\gamma_{i}={\left({\tilde{T}}_{i}\cdot E_{i,2}\right)}^{2}$, $\beta_{i}=({\tilde{T}}_{i}\cdot E_{i,1})({\tilde{T}}_{i}\cdot E_{i,2})$. The tangential shear stress across the surface curve with unit normal ***ν***_*i*_ = cos*θ****E***_*i*,1_ + sin*θ****E***_*i*,2_ is given by the expression

$$\nu_{i}^{⊥}\cdot {\tilde{T}}_{i}\nu_{i}=\beta_{i}\cos(2\theta )+\frac{1}{2}(\gamma_{i}-\alpha_{i})\sin(2\theta ),$$

where $\nu_{i}^{⊥}=-\sin\theta E_{i,1}+\cos\theta E_{i,2}$. Then the maximum shear stress is computed as

$$\tau_{\max,i}=\frac{1}{2}\sqrt{4\beta_{i}^{2}+{\left(\gamma_{i}-\alpha_{i}\right)}^{2}},$$

coinciding with the expression given in [6,8] for *α*_*i*_ = *γ*_*i*_. The direction of the line across which the tangential shear stress is maximal is defined by the angle *θ*_max,*i*_ such that:

(i) for *α*_*i*_ < *γ*_*i*_, *β*_*i*_ > 0, $\theta_{\max,i}=\frac{1}{2}\arctan((\gamma_{i}-\alpha_{i})/2\beta_{i})$;
(ii) for *α*_*i*_ < *γ*_*i*_, *β*_*i*_ < 0, $\theta_{\max,i}=\frac{1}{2}\arctan((\gamma_{i}-\alpha_{i})/2\beta_{i})\;+$ (π/2);
(iii) for *α*_*i*_ > *γ*_*i*_, *β*_*i*_ > 0, $\theta_{\max,i}=\frac{1}{2}\arctan((\gamma_{i}-\alpha_{i})/2\beta_{i})+\pi$ ;
(iv) *α*_*i*_ > *γ*_*i*_, *β*_*i*_ < 0, $\theta_{\max,i}=\frac{1}{2}\arctan((\gamma_{i}-\alpha_{i})/2\beta_{i})+(\pi /2)$.

**A.4. Invariance under icosahedral symmetry**

We prove here that, under suitable assumptions on the interaction energy, the lateral stress and the maximum tangential shear stress have icosahedral symmetry as specified below.

We say that a configuration *X* is icosahedral if it is invariant under the icosahedral group I⊂SO(3). In this case, we can characterize the configuration as the union of orbits of I. The action of I can then be expressed in terms of a permutation representation as follows. For every Q∈I, there exists a permutation *ρ* on *N* elements such that

A 1 $$Qx_{i}=x_{\rho (i)},\;\forall i=1,\ldots ,N.$$

We write this permutation representation as $I\to \Sigma \subset S_{N}$, with *S*_*N*_ the permutation group on *N* elements. Note that the 12 distinguished points at the fivefold axes representing the pentamers must belong to a single orbit.

Let *V*_*ij*_(*r*_*ij*_) be an interaction energy between points ***x***_*i*_ and ***x***_*j*_, depending only on their distance (not necessarily the Lennard-Jones energy (2.1)), and assume that it is icosahedrally invariant, i.e.

$$V_{\rho (i)\rho (j)}(r_{\rho (i)\rho (j)})=V_{ij}(r_{ij}),\;\forall i,j=1,\ldots ,N\;\text{and}\;\forall \rho \in \Sigma ,$$

which, given (A 1), is equivalent to the simpler requirement

A 2 $$V_{\rho (i)\rho (j)}(r)=V_{ij}(r),\;\forall i,j=1,\ldots ,N\;\text{and}\;\forall \rho \in \Sigma ,$$

with *r* ∈ (0, + ∞). Then the Lennard-Jones energy (2.1) is icosahedrally invariant.

We now show that the Cauchy stress is invariant under the icosahedral group, in the sense that, for every icosahedrally invariant configuration *X*, and every Q∈I, with associated permutation *ρ*,

A 3 $$T_{\rho (i)}(X)=QT_{i}(X)Q^{⊤}.$$

In fact, letting $\bar{\rho }$ be the inverse permutation of *ρ*, using (A 2), (A 1) and the fact that the icosahedral group is orthogonal, we obtain

$$\begin{array}{rl} T_{\rho (i)}(X) & =-\frac{1}{2}\sum_{\;j\neq \rho (i)}\frac{1}{r_{\rho (i)j}}\frac{\text{d}V_{\rho (i)j}}{\text{d}r}(r_{\rho (i)j})r_{\rho (i)j}⊗r_{\rho (i)j}\\ & =-\frac{1}{2}\sum_{\;j:\bar{\rho }(j)\neq i}\frac{1}{r_{i\bar{\rho }(j)}}\frac{\text{d}V_{i\bar{\rho }(j)}}{\text{d}r}(r_{i\bar{\rho }(j)})Qr_{i\bar{\rho }(j)}⊗Qr_{i\bar{\rho }(j)}\\ & =-\frac{1}{2}\sum_{\;j\neq i}\frac{1}{r_{ij}}\frac{\text{d}V_{ij}}{\text{d}r}(r_{ij})Qr_{ij}⊗Qr_{ij}, \end{array}$$

which is equivalent to (A 3).

An immediate consequence of (A 3) is that all orthogonal invariants of the stress are also icosahedrally invariant. This, in turn, implies that

$$\Lambda_{\rho (i)}=\Lambda_{i},\;\tau_{\max,\rho (i)}=\tau_{\max,i},\;\forall \rho \in \Sigma .$$

A second consequence of icosahedral invariance is that pentamers cannot support shear stresses, since $\tilde{T}$ is proportional to the identity tensor, so that the eigenvalues of $\tilde{T}$ are equal and the maximum tangential shear stress vanishes. The same conclusion holds if there are points (hexamers) at threefold axes. Both assertions follow from the elementary fact that, if ***Q******x***_*i*_ = ***x***_*i*_ for some ***Q***, i.e. if ***Q*** is in the isotropy group of ***x***_*i*_, then $Q{\tilde{T}}_{i}=\tilde{T}Q$, and the only 2 × 2 matrices commuting with three- and fivefold rotations are proportional to the identity.

This result implies that the shear stress cannot concentrate at three- and fivefold axes. Note also that the components *α*, *β* and *γ* of the tangential stress with respect to a given basis are not icosahedrally invariant.
